# Theoretical Modeling and Experimental Detection of the Extracellular Phasic Impedance Modulation in Rabbit Hearts

**DOI:** 10.3389/fphys.2019.00883

**Published:** 2019-07-09

**Authors:** Shahriar Iravanian, Conner Herndon, Jonathan J. Langberg, Flavio H. Fenton

**Affiliations:** ^1^Division of Cardiology, Emory University, Atlanta, GA, United States; ^2^School of Physics, Georgia Tech, Atlanta, GA, United States

**Keywords:** cardiac electrophysiology, ionic channels, impedance, membrane biophysics, computational biology

## Abstract

Theoretical cardiac electrophysiology focuses on the dynamics of the membrane and sarcoplasmic reticulum ion currents; however, passive (e.g., membrane capacitance) and quasi-active (response to small signals) properties of the cardiac sarcolemma, which are quantified by impedance, are also important in determining the behavior of cardiac tissue. Theoretically, impedance varies in the different phases of a cardiac cycle. Our goal in this study was to numerically predict and experimentally validate these phasic changes. We calculated the expected impedance signal using analytic methods (for generic ionic models) and numerical computation (for a rabbit ventricular ionic model). Cardiac impedance is dependent on the phase of the action potential, with multiple deflections caused by a sequential activation and inactivation of various membrane channels. The two main channels shaping the impedance signal are the sodium channel causing a sharp and transient drop at the onset of action potential and the inward rectifying potassium channel causing an increase in impedance during the plateau phase. This dip and dome pattern was confirmed in an *ex-vivo* rabbit heart model using high-frequency sampling through a monophasic action potential electrode. The hearts were immobilized using a myosin-inhibitor to minimize motion artifacts. We observed phasic impedance changes in three out of four hearts with a dome amplitude of 2 − 4Ω. Measurement of phasic impedance modulation using an extracellular electrode is feasible and provides a non-invasive way to gain insight into the state of cardiac cells and membrane ionic channels. The observed impedance recordings are consistent with the dip and dome pattern predicted analytically.

## 1. Introduction

Theoretical cardiac electrophysiology focuses on the dynamics of the membrane and sarcoplasmic reticulum ion currents that are voltage, time, and ligand-dependent. Conversely, the passive electrical properties are usually relegated to a secondary role and are simplified as a constant membrane capacitance (usually assumed to be 1μ*F*/cm^2^) and constant intracellular and extracellular bulk conductance. In the words of Silvio Weidmann, one of the founders of cardiac electrophysiology, “A knowledge of the myoplasm resistance and the membrane resistance and capacity is important in discussions of cardiac excitation and conduction” (Niggli et al., 2006). The membrane resistance, or more correctly the transmembrane impedance, is dependent on the state of the membrane ion channels. This dependence and its practical measurement are the main subjects of this paper.

The origin of this paper was an attempt to answer a seemingly simple question: what type of impedance signal is measured if we place an extracellular electrode near cardiac tissue and pass a test signal (e.g., a sinusoidal current) to measure the extracellular impedance during a cardiac cycle? As we will see, myocardial impedance is rather complicated with both frequency and phase dependency, where phase refers to different stages of an action potential.

Myocardial impedance patterns are relevant to clinical electrophysiology as well. Intra-cardiac electrograms are the cornerstone of electrophysiology. For example, local peak-to-peak voltage is used to characterize tissue, e.g., to differentiate healthy cardiac tissue from fibrosis (Zeppenfeld et al., 2005). However, the mere voltage signal is not always sufficient and, for many applications, something more is needed. Impedance is technically easy to measure and may provide additional information.

As we will show in the next section, the membrane impedance has three main components: the membrane capacitance, which is generally considered constant throughout a cardiac cycle, the static impedance, which is dependent on the state of ion channels at each point in time, and the dynamic impedance, caused by the perturbation of the ionic currents by the test signal. The reason for a dynamic impedance response is the presence of voltage dependent ionic currents. Assume that we deliver an excitatory (depolarizing) current pulse to a quiescent (resting) cardiac cell. The transmembrane potential (*V*_*m*_) increases (becomes less negative). As a result, a few sodium channels open. The inward current through the sodium channels further depolarizes the cell. A positive feedback loop is formed and generates an action potential or, in the language of non-linear dynamics, a subcritical Hopf bifurcation occurs (Izhikevich, 2010). This is the classic *active* membrane response. Now, let's look at a sub-threshold pulse. Again, some sodium channels open as a result of the change in *V*_*m*_; however, this time the amplification ratio is less than 1 and no runaway reaction ensues. Nonetheless, the opening of sodium channels changes the membrane impedance. Therefore, we cannot simply assign a constant impedance value to the membrane. Instead, we need to apply a *linearization* procedure to the ionic model to find the phase specific impedance. The linearization process was applied to the Hodgkin-Huxley model in 1960s and 1970s (Fitzhugh, 1962; Sabah and Leibovic, 1969; Mauro et al., 1970). Koch called such an impedance response to sub-threshold stimulation quasi-linear and investigated it in relation to single-neuron computations (Koch, 1984).

The discovery of impedance drop at the onset of action potentials was a significant milestone in elucidating the mechanisms of action potential generation and was critical in formulating the Hodgkin-Huxley model (Cole and Curtis, 1939; Hille, 2001). In 1951, Weidmann measured the impedance changes in the course of the cardiac cycle in mammalian hearts (goat Purkinje fibers) using an intracellular electrode and confirmed the significant drop in impedance at the onset of action potentials (Weidmann, 1951). He also reported that the measured resistance during the plateau was comparable to that in diastole and increased as the phase of repolarization was approached. Spitzer et al. investigated the interactions between membrane and gap junctional resistance in different phases of the action potential and noted that these interactions may affect cardiac conduction and arrhythmogenicity (Spitzer et al., 2006).

In this paper, we first develop a theoretical framework and show both analytically and numerically the expected phasic impedance modulation. Afterward, we validate the modeling results with *ex-vivo* measurement of impedance signals in rabbit hearts using an extracellular electrode and show that the detection of phasic impedance modulation is feasible.

## 2. Theoretical Modeling

In this section, we start by calculating the expected membrane impedance of a single cardiac cell as measured by an intracellular electrode (denoted by *Z*). Based on the knowledge of the intracellular impedance, we then calculate the extracellular impedance, which is shown as ℤ.

### 2.1. Impedance Calculation for Generic Ionic Models

The starting point is a generic cardiac ionic model

(1) $$C\frac{dV}{dt}=\;-I(V,\;f_{1},\;f_{2},\ldots ,f_{n};P)+I_{\text{ext}},$$

where *V* is the transmembrane potential (we have dropped the customary *m* subscript for simplicity), *C* is the membrane capacitance, *f*_*i*_s are *n* Hodgkin-Huxley style gating variables, **P** is a collection of other state variables (e.g., ionic concentrations) which are assumed constant for this analysis, *I*() is the sum of the transmembrane currents, and *I*_ext_ is the injected current (e.g., the test signal).

The dynamics of the gating variables is described as

(2) $$\begin{array}{r} \frac{df_{i}}{dt}=-\frac{1}{\tau_{i}\left(V\right)}\left[f_{i}-f_{i}^{eq}\left(V\right)\right]. \end{array}$$

where $f_{i}^{eq}$ and τ_*i*_ are the steady-state (equilibrium) value and time-constant of *f*_*i*_, respectively.

The general formula for the (frequency-dependent) impedance of an isolated myocardium or a patch of sarcolemma is derived in Supplement A and is

(3) $$\begin{array}{r} \frac{1}{Z\left(\omega \right)}=\frac{\partial I}{\partial V}-i\omega C+\underset{i}{\sum }\left(\frac{1}{1-i\omega \tau_{i}\left(V\right)}\frac{df_{i}^{eq}\left(V\right)}{dV}\frac{\partial I}{\partial f_{i}}\right), \end{array}$$

where *Z* is the impedance and ω is the angular frequency of the test signal, e.g., a sinusoidal current injected into an electrode to measure impedance.

Equation (3) is the cornerstone of impedance calculation. The first term, ∂*I*/∂*V*, is *conductance*; i.e., the first-order approximation to admittance (the inverse of impedance) based on the state of the ionic channels irrespective of the test signal (the static impedance). The second term, −*iωC*, is the *susceptance* (the inverse of reactance) due to the membrane capacitance.

The third term represents the dynamic impedance. If for a given *f*_*i*_, the time-constant is small such that $\tau_{i}\left(V\right)\ll \omega^{-1}$, then we have ${\left(1-i\omega \tau_{i}\left(V\right)\right)}^{-1}\approx 1$. Therefore, for this gating variable, the third term reduces to the chain-rule of differentiation and acts as conductance only. However, if $\tau_{i}\left(V\right)\gg \omega^{-1}$, then the leading factor is ${\left(-i\omega \tau_{i}\left(V\right)\right)}^{-1}$, which behaves like inductance. This *phenomenological inductance* is responsive, among others, for the hyper-polarization tail of a sub-threshold depolarizing pulse (Cole, 1941; Cole and Baker, 1941). Intuitively, this inductance behavior is caused by slow channels (relative to the test signal) that lag behind the transmembrane potential. The interaction of the membrane capacitance and pseudo-inductance forms an RLC circuit (Koch, 2004; Felton et al., 2018) that can exhibit *resonance* phenomena.

### 2.2. Impedance of the Mahajan Rabbit Ventricular Model (Intracellular Electrode)

Let's apply the generic formula for the calculation of admittance to a concrete cardiac model. We have chosen the Mahajan-Shiferaw rabbit ventricular model (the Mahajan model from here on) as our example (Mahajan et al., 2008). The data presented in the results section were collected from rabbit hearts; therefore, a rabbit model allows us to correlate the experimental data with the simulated impedance. The Mahajan model is a modified Shannon rabbit model (Shannon et al., 2004) with improved calcium handling. It has 26 state variables: the transmembrane potential (*V*), 10 Hodgkin-Huxley style gates, 7 intracellular ionic concentrations and currents, 2 troponin buffers, and 6 variables describing the Markov model formulating the L-type calcium channel. The ten gating variables correspond to the *f*_*i*_s in Equations (1)–(3).

In classic papers about the impedance of ionic models (Fitzhugh, 1962; Sabah and Leibovic, 1969; Mauro et al., 1970; Koch, 1984), the equations for ionic currents were substituted in Equation (3) or its counterparts and analytically expanded. The resulting equations can easily become too complicated and are not very informative. The analytic route is only applicable to simple ionic models (mainly the Hodgkin-Huxley model). In this paper, we solved this equation directly with the help of automatic-differentiation using dual numbers. The Julia programming language, which is particularly suitable for automatic-differentiation, was used (Revels et al., 2016; Bezanson et al., 2017).

**Figure 1:**
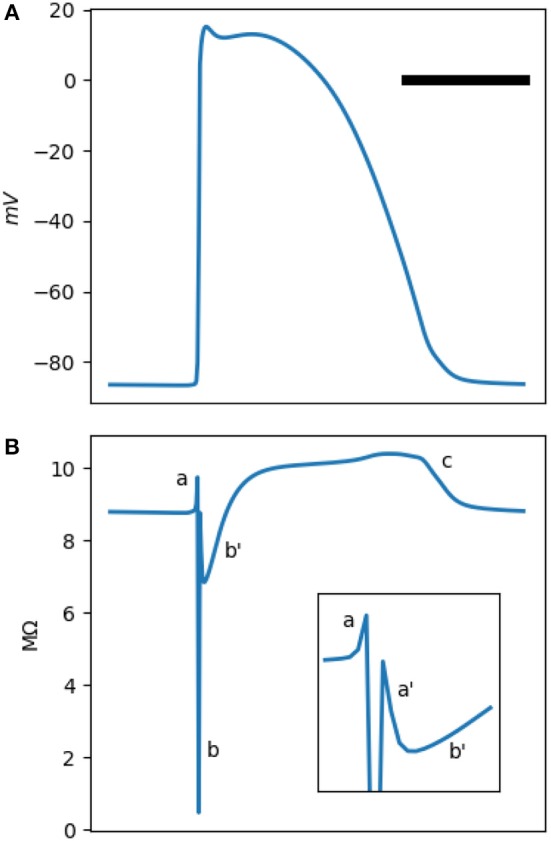
**(A)** is a typical Mahajan rabbit ventricular action potential. The horizontal bar represents 100 ms. **(B)** The phasic changes in the impedance of a standard Mahajan ventricular cell ($C_{m}=3.1\times 10^{-4}\mu F$). The inset zooms in the period around the onset of the action potential. Different impedance deflections (a,a',b,b',c) are marked. See the main text for the description of each one. The impedance was calculated at a test frequency of 50 Hz.

**Figure 2:**
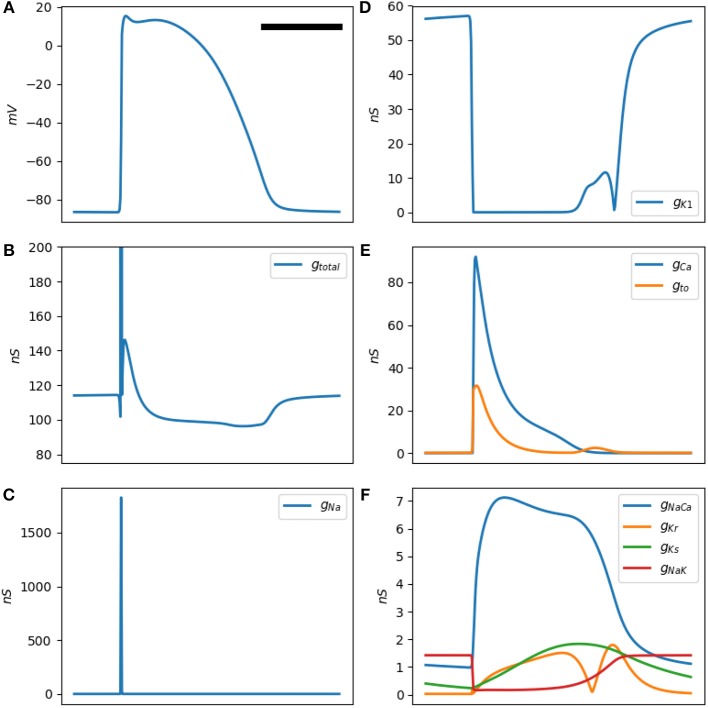
**(A)** is a typical Mahajan rabbit ventricular action potential. The horizontal bar represents 100 ms. **(B)** The total membrane conductance (this figure is the inverse of **(B)**. **(B–F)** Conductance of various ionic channels at a test frequency of 50 Hz for a standard Mahajan ventricular cell ($C_{m}=3.1\times 10^{-4}\mu F$). The y-axis unit is in *nS*, which stands for nanosiemens.

Figures 1A, 2A show a typical simulated rabbit ventricular action potential. The Mahajan model equations were integrated using the explicit Euler method with a semi-closed form for the Hodgkin-Huxley variables, the Rush-Larsen technique (Rush and Larsen, 1978), and a time step of 0.01 ms.

The impedance of a single cell, as measured by an intracellular electrode, is depicted in Figure 1B. The impedance signal has multiple positive and negative deflections. We identify five deflections. There is a small positive (impedance increasing) deflection (**a**) coincidental with the upstroke of the action potential, followed by a sharp and deep negative wave (**b**). The impedance drops to less than a tenth of its baseline value in **b**. Another small positive deflection, (**a'**), follows **b**. There are two impedance deflections during the plateau of the action potential. A shallow and slow drop (**b'**) and then a gradual rise in the impedance that peaks during phase 3 of the action potential (**c**).

The **a-b-a'-b'-c** pattern is generated by the activation and inactivation of various membrane currents during the time course of the action potential. Since the membrane currents form a parallel network, it is easier to reason in term of conductance instead of impedance. Figure 2B shows the total cellular conductance, corresponding to Figure 1B, which is decomposed to its constituent conductances in Figures 2C–F. It is obvious that the sodium current is responsible for the transient and deep increase in conductance at the onset of the action potential (the **b** wave, Figure 2C). This wave correlates with the impedance drop of the squid giant nerves discovered at the dawn of electrophysiology (Cole and Curtis, 1939).

The other ionic current that has a significant role in shaping the conductance is the inward rectifying potassium current *I*_*K*1_ (Figure 2D). This current is responsible for the resting membrane potential. *I*_*K*1_ is voltage-dependent and has a very low conductance when the transmembrane potential is positive. In fact, its conduction begins to drop even before the sodium current reaches the threshold and activates; hence, the **a** wave. After the sodium current inactivates, *I*_*K*1_ still has a lower than baseline conductance and generates **a'**. The L-type calcium and the transient outward potassium (*I*_*to*_) currents become active in the early segment of the plateau (wave **b**, Figure 2E). In the later part of the plateau and phase 3 of the action potential, these currents are mostly inactive; while the decrease in the *I*_*K*1_ conductance persists and manifests as **c**. A multitude of other currents have a low peak conductance and do not significantly contribute to the shape of the impedance signal (Figure 2F).

**Figure 3:**
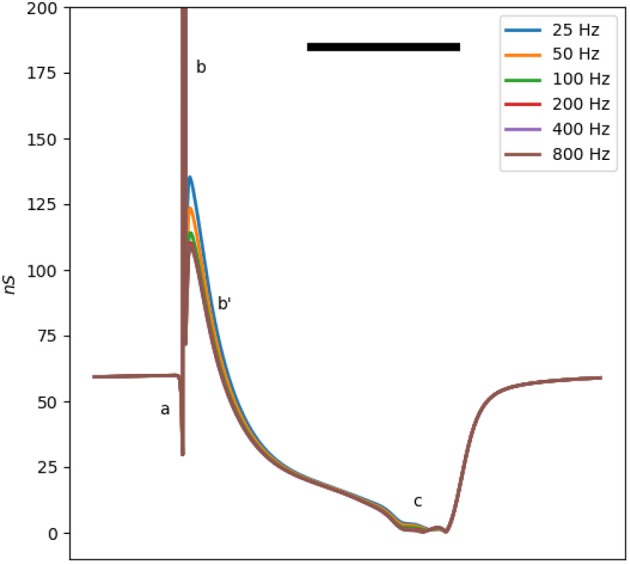
The total membrane conductance of the Mahajan rabbit ventricular model at different test signal frequencies derived using Equation (3). The effect of capacitance is removed to ease comparison. Different impedance/conductance deflections (**a**,**b**,**b'**,**c**) are marked, similar to Figure 1. Note that the main difference is in the **b'** wave, caused by the *I*_*to*_ and *I*_*Ca, L*_ currents. The horizontal bar represents 100 ms.

The membrane impedance is frequency-dependent. The ωτ_*i*_ term in Equation (3), corresponding to the membrane capacitance, confers a linear dependency of the conductance on frequency. Moreover, the third term in Equation (3) also depends on the frequency through the ${\left(1-i\omega \tau_{i}\left(V\right)\right)}^{-1}$ factor. Figure 3 shows the conductance of a single Mahajan cell after the removal of the capacitance effect at different test signal frequencies (20–800 Hz). For this range of test frequencies, the main difference is in **b**, where the conductance increases as the test frequency decreases.

### 2.3. Impedance of the Mahajan Rabbit Ventricular Model (Extracellular)

**Figure 4:**
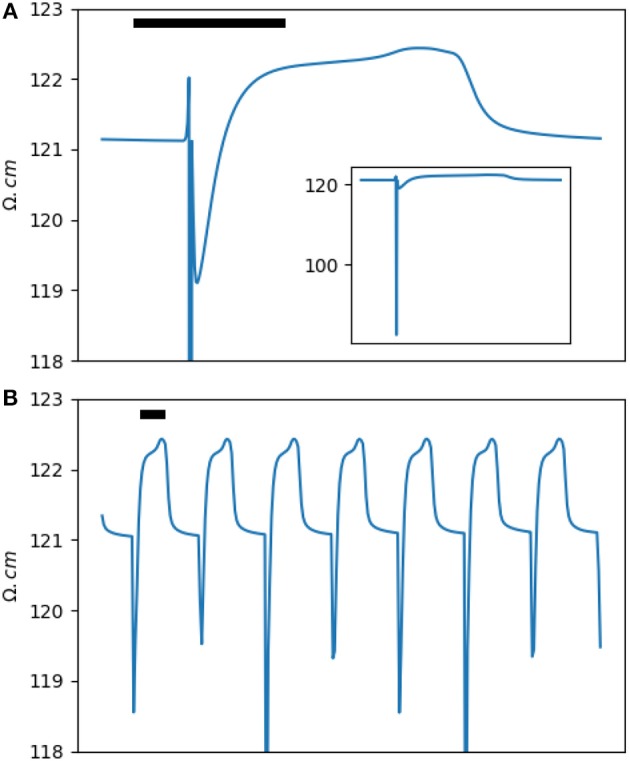
**(A)** The extracellular impedance of a two-dimensional rabbit ventricular model. The inset shows a full scale version of the extracellular impedance. **(B)** The impedance signal down-sampled to a sampling rate of 100 Hz (corresponding to a window of 10 ms) to simulate the experimental results. Note the dip and dome pattern. The horizontal bar represents 100 ms.

The impedance analysis presented in the previous section dealt with the impedance measured using an intracellular electrode in a single cell. However, cardiac tissue is a syncytium. Analytic solutions to the apparent intracellular impedance for the cable models and two- and three-dimensional cardiac tissue are available (Eisenberg and Johnson, 1970; Jack et al., 1983; Koch, 2004; Plonsey and Barr, 2007). Additionally, in this paper, we are mainly interested in the extracellular impedance. The extracellular space provides a low impedance pathway that shunts most of the injected current and blunts the amplitude of the impedance phasic changes. In Supplement B, the extracellular impedance of a two-dimensional segment of cardiac tissue and its phasic modulation are derived using a semi-analytic method based on the bidomain methodology (Figure 4A). The result is essentially a scaled version of the intracellular impedance.

Let's define the amplitude of the impedance changes, Δ*Z* or Δℤ, as the difference between the peak impedance in phase 2/3 of an action potential to the resting impedance (ignoring the transient dip in phase 0). The relative intracardiac impedance changes, Δ*Z*/*Z*, is around 15% and is significantly larger than the relative extracellular impedance changes, Δℤ/ℤ, of less than 1%.

In the results section, we will present impedance measurements in *ex-vivo* rabbit hearts. As we will see, for theoretical and practical reasons, impedance sampling was done in 10 ms windows. Figure 4B shows the same data as in Figure 4A down-sampled to 10 ms. Comparing the two figures, we notice that the **a** and **a'** waves are not seen anymore and the two **b** and **b'** waves are merged into a single dip of variable depth. The **b/b'** dip and the **c** impedance rise form a *dip and dome* pattern. Experimentally, our main goal was to detect and document the dip and dome signature in cardiac tissue.

## 3. Experimental

### 3.1. Methods

#### 3.1.1. Experimental Preparation

All experiments conform to the current Guide for Care and Use of Laboratory Animals published by the National Institutes of Health (NIH Publication No. 85-23, revised 1996) and approved by the Office of Research and Integrity Assurance at Georgia Tech.

New Zealand White Rabbits (2–3 kg, *n* = 4) of either sex were anesthetized with intra-peritoneal ketamine/xylazine/acepromazine (17/9/0.9 mg/kg). After 15 min, isoflurane (1–3% delivered in oxygen) was administered to achieve deep anesthesia. The animals were injected with heparin (300 U/kg). Following euthanasia via bilateral thoracotomy, hearts were quickly perfused retrogradely through the aorta with Tyrode's solution [in g/L: NaCl 7.24, KCl 0.3, NaHCO 3 2.02, NaH 2PO 4·H 2O 0.12, MgCl·6H2O 0.14, CaCl 2·2H 2O 0.29, dextrose 0.99] until coronary arteries were cleared of blood and were then perfused with cardioplegic solution [in g/L: NaCl 6.42, KCl 1.19, NaHCO3 0.84, MgCl·6H2O 3.25, CaCl2 0.13] for transport.

Once in the lab, hearts were mounted on a Langendorff apparatus and perfused with Tyrode's solution gassed with 95 O2 and 5% CO2 warmed to 38 ± 1°C and maintained at a perfusion pressure of 60 mmHg by a peristaltic pump. Cardiac motion was suppressed by adding 3–5 mM of myosin-inhibitor blebbistatin dissolved in dimethyl sulfoxide (DMSO) (Fedorov et al., 2007). The experiments were done as part of optical mapping sessions. Therefore, the hearts were also stained with 0.4 mg of fluorescent voltage-sensitive dye JPW-6003 dissolved in 40 μL of pure ethanol.

#### 3.1.2. Impedance Measurement

We recorded monophasic action potentials (MAP) and impedance signals with the help of a two-electrode impedance measurement setup. The MAP electrode was made of a silver wire (1.6 mm in diameter) insulated except at one end, where a bevel was cut at a 45° angle to provide a sharp but relatively non-traumatic tip. Both the recording electrode, pushing against the left ventricular epicardium, and the reference electrode in the bath were covered with silver chloride to improve their low-frequency characteristics.

Direct Digital Synthesis (DDS) technique was used to generate sinusoidal test signals with a frequency range of 2–2,000 Hz. DDS was implemented using a Field Programmable Gated Array (FPGA) connected to a neurophysiology amplifier (RHD2000, Intan technologies, Los Angeles, CA). A custom-made analog head-stage conditioned and pre-amplified the test and voltage signals. The system was configured to generate and acquire signals at 30 KHz and with a resolution of 16 bits.

The reason that we used a MAP electrode to measure impedance instead of a microelectrode or a standard electrophysiology catheter is that MAP electrodes have low impedance and form a tight coupling to the myocardium. A typical electrophysiology catheter has a much larger electrode surface area and leaks most of the injected current to the bath. On the other hand, microelectrodes commonly used in electrophysiology research are high-impedance and are unsuitable in a two-electrode setup.

#### 3.1.3. Signal Processing

The head-stage injected a test sinusoidal current *I*_0_sin(ω*t*), for *I*_0_ = 0.01−0.03mA, into the MAP electrode and recorded the resulting voltage response, say *y*(*t*). Both *y*(*t*) and the instantaneous test signal phase, ϕ = ω*t*, were transmitted to a computer for offline processing.

Each voltage recording was processed to obtain both impedance and MAP signals. The voltage signal, *y*(*t*), was divided into non-overlapping 10 ms windows and impedance was calculated for each segment independently. The impedance signal has the same frequency as the test signal and can be separated from the electrogram by the help of frequency analysis. However, there is a potential overlap between the impedance and MAP signals in the frequency domain that can cause cross-talk. To minimize this overlap, the processing should be optimal (in the least-square sense) with a sharp frequency selection and minimum sidebands. This was achieved by using the *Lomb-Scargle* spectrogram method, as detailed in Supplement C.

A Wiener filter processed the raw recordings to generate the MAP signals. Control recordings with no test signal were used as the reference for the Wiener filter.

The choice of a 10 ms sampling window allowed for an acceptable reconstruction of the phasic impedance modulation. In turn, the length of the sampling window affected the choice of the test signal frequency. The membrane capacitance dominates the membrane conductance at high test frequencies, such that the contribution of ionic channels in the total conductance decreases and becomes difficult to detect. On the other hand, to ensure an acceptable frequency resolution, the sampling window should be large enough to contain at least 5 cycles of the test signal. A test signal frequency of 500–1,000 Hz satisfies both conditions.

### 3.2. Results

We measured impedance in four rabbit hearts and detected phasic changes in three cases with a Δℤ of 2–4 Ω at 1,000 Hz. In the fourth case, tight coupling between the electrode and tissue was not formed as a result of a suboptimal electrode and Δℤ was small (0.5 Ω), which is comparable to the noise level in our system and could not be unequivocally confirmed.

**Figure 5:**
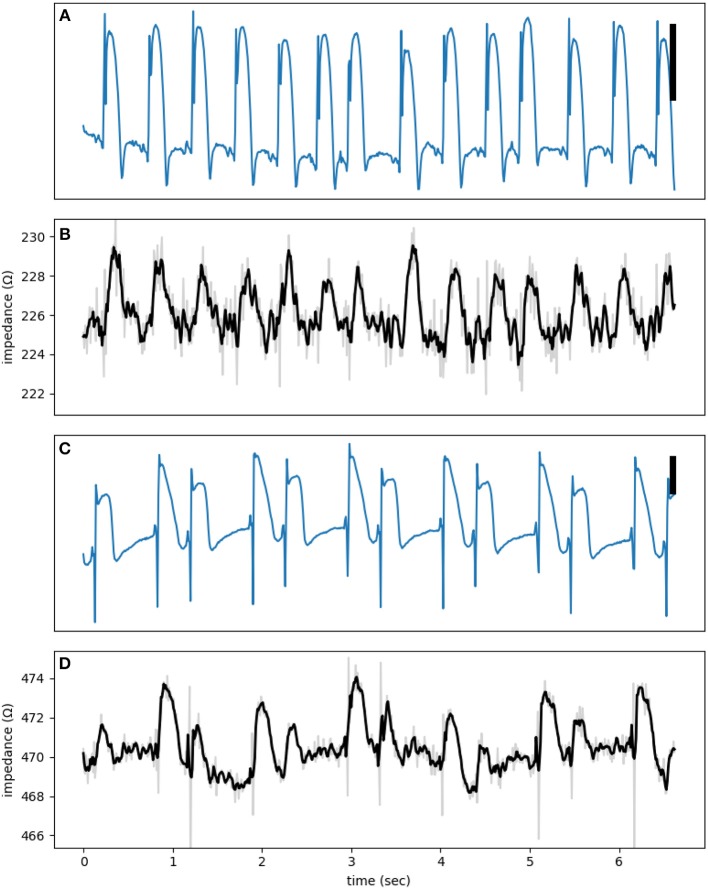
**(A,C)** are electrograms showing MAP signals with **(B,D)** the corresponding impedance signals at a test signal frequency of 1,000 Hz. The raw impedance signals (gray) are smoothed using a third order Savitzky-Golay filter (black lines). The vertical bar represents 10 mV.

Figure 5 shows two examples of phasic impedance changes. Impedance drops transiently at the onset of action potentials then increases during phase 2 with a return to baseline in phase 3. This pattern is consistent with the dip and dome pattern predicted from the theoretical and numerical works.

**Figure 6:**
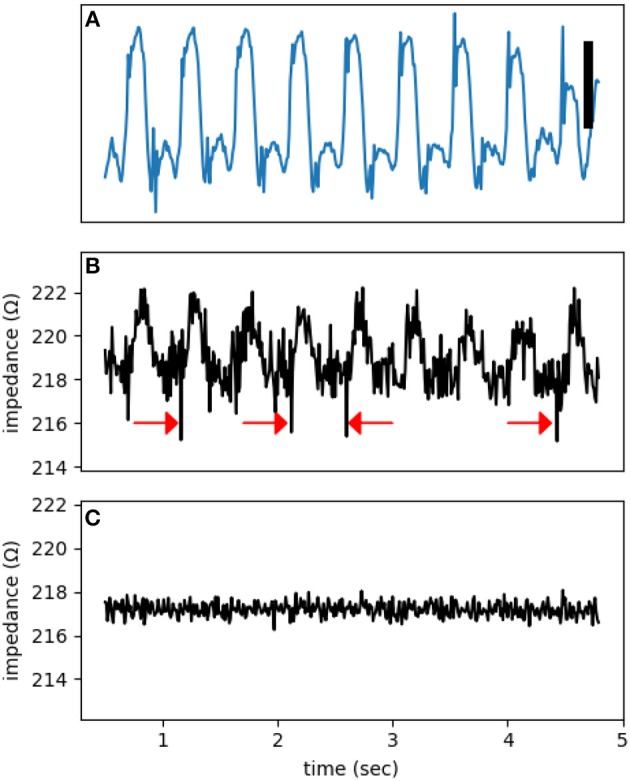
A representative electrogram **(A)** and impedance modulation **(B)**. Note the impedance dips coincide with the onset of action potentials (red arrows). **(C)** A control impedance signal using a synthetic input based on **(A)**. The test signal frequency was at 1,000 Hz. The vertical bar represents 10 mV.

The dome is obvious, but the dip is a transient phenomenon. The activation of sodium channels, forming the bulk of the dip, lasts less than 1 ms and can be missed or averaged out in an impedance measurement window of 10 ms (the *aliasing* phenomenon). Figure 6B shows multiple dips that coincide with the upstroke of action potentials. To claim that these observed dips are true impedance signal, we need to exclude potential confounding sources. The main artifact at high frequencies relevant to the detection of dips is cross-talk from the MAP channel into the impedance signals. By using synthetic signals, we can put an upper bound on the magnitude of the cross-talk. To generate Figure 6C, we started from the signal in Figure 6A, added an unmodulated sinusoidal signal, and then subjected it to the same processing as in Figure 6B. Lack of dips in Figure 6C is reassuring that the observed dips in Figure 6B represent true impedance changes.

**Figure 7:**
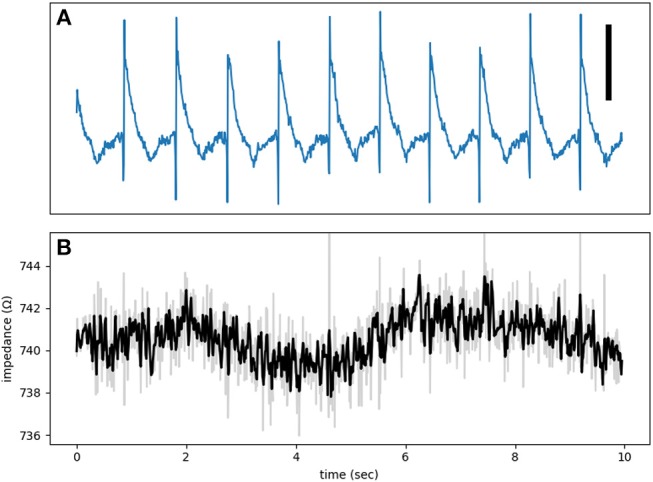
**(A)** An electrogram recorded while the electrode was touching, but not forcing into, the tissue. The signal is an example of a unipolar recording and not a true MAP. **(B)** The impedance signal shows no obvious phasic modulation. The test signal frequency was at 1,000 Hz. The vertical bar represents 10 mV.

Additional evidence against the presence of significant cross-talk is provided by control recordings. Figure 7 is from such a control run when the electrode was touching but not pushing against the heart. As a result, the electrogram shows a unipolar recording instead of a typical MAP signal. There is no discernible impedance modulation and the impedance signal is essentially all noise.

**Figure 8:**
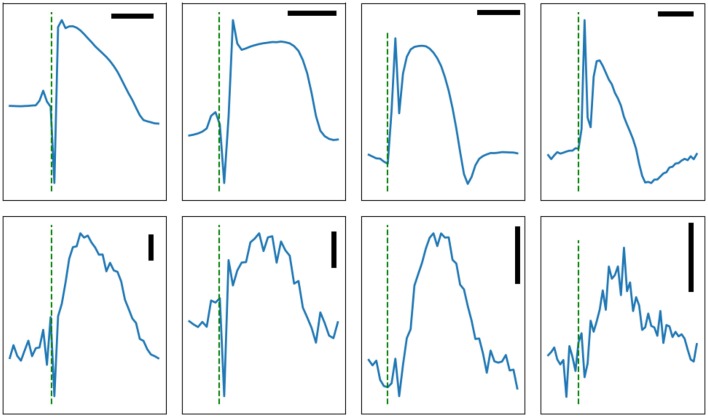
Four signal-averaged MAP (top row) and the corresponding impedance signals (bottom row) recorded from rabbit left ventricles, except for the right column which shows atrial signal. The horizontal bars are 100 ms in duration. The vertical bars represent 1Ω. The test signal frequency was at 1,000 Hz except for the rightmost column where it was 500 Hz. Note the presence of obvious dips on the two leftmost panels.

For the dome, which is generally a low-frequency feature, the main potential artifact is myocardial motion. For these experiments, the hearts were infused with a myosin-inhibitor and were visually paralyzed. However, some residual microscopic motions cannot be excluded. Motion artifacts are usually symmetrical with a late peak compared to action potentials and a return to baseline after phase 3; i.e., the motion artifact has a significant diastolic component caused by the relaxation phase. Figure 8 shows multiple representative signal-averaged impedance signals. The dome is well contained within the plateau of action potentials, as expected of a true impedance signal.

## 4. Discussion

In this paper, we report the detection of phasic impedance modulation as measured by an extracellular electrode in close proximity of cardiac tissue. The observed dip and dome pattern correlates well with the analytic and modeling results presented in the first part of the paper. This method opens the dynamics of the membrane ionic channels to non-invasive extracellular inspection.

The amplitude of the phasic changes in the extracellular impedance is relatively small (few ohms compared to few hundred ohms for the total impedance). The detection of such small impedance modulation is experimentally feasible but requires careful attention to the details, such as the dimensions and characteristics of the recording electrode, the frequency range of the test signal, and proper signal processing.

The membrane impedance and its accompanying features, such as pseudo-active response and membrane resonances, are well-known in theoretical neurophysiology literature but are generally absent from the contemporary cardiac studies. For example, the frequency sensitivity of vestibular hair cells is attributed to membrane resonance (Hudspeth and Lewis, 1988a,b). Another example is the spatiotemporal tuning of retinal ganglionic cells (Derrington and Lennie, 1982; Enroth-Cugell et al., 1983). The cortical pyramidal cells have different membrane resonance zones, which are important in information processing and communication between neurons (Felton et al., 2018). Whether such pseudo-active features have any physiological or pathological role in the heart is unknown but possible. For example, they may contribute to the generation of early afterdepolarization or synchronization of activity in coupled pacemaker cells (as in the sinus node).

The established technique of impedance cardiography measures the cardiac output from the variations of thoracic impedance, which depends on the instantaneous volume of the intrathoracic blood and the differential conductivity of blood and tissues (Kubicek et al., 1966). Another application of impedance measurement in cardiology is the assessment of tissue hypoxia. The basis of this method is hypoxia-induced cell-to-cell decoupling (Kléber et al., 1987; Cascio et al., 1990). In contrast to these methods, we are primarily interested in the *phasic* changes in the *intrinsic* impedance of cardiac tissue, regardless of any motion or change in geometry.

Beyond its theoretical importance, measurement of phasic impedance changes has practical benefits. Extracellular impedance measurement is technically accessible, is not harmful to cells, and can continue for a long time. There are hundreds of cardiac ionic models, from simple ones like the Hodgkin-Huxley model with three gates to complex ones with up to 30–40 state variables (Fenton and Cherry, 2008). A complex model can easily have hundreds of parameters that need to be tuned and adjusted. This raises the possibility of over-fitting that can seriously limit the generality and predictive power of these models. Extracellular impedance can act as a measure of the global goodness of fit of a model to shrink the parameter space and exclude unrealistic parameter values that may otherwise be selected.

As mentioned in the Introduction, the original motivation for this study was to find a practical way to assess the state of cardiac tissue during clinical electrophysiology studies. The results are encouraging that phasic impedance changes can be useful in this regard. However, there are practical challenges in realizing this goal. The biggest challenge is motion-induced impedance variations, which are at least an order of magnitude larger than what we have measured here. In this study, hearts were immobilized by blebbistatin. Of course, a myosin inhibitor cannot be used clinically! We expect that, by combining multi-frequency measurements and a multi-electrode setup to measure and subtract motion artifacts, it is possible to detect impedance modulation in a clinical setting.

Since membrane impedance is an aggregate measure of the state of ionic channels, interventions that affect ionic channels, such as membrane active antiarrhythmic medications, may also alter the impedance pattern. Therefore, it is plausible that by measuring phasic impedance modulation one may evaluate the state of ionic channels and response to medications.

## Data Availability

The datasets generated for this study are available on request to the corresponding author.

## Ethics Statement

All experiments conform to the current Guide for Care and Use of Laboratory Animals published by the National Institutes of Health (NIH Publication No. 85-23, revised 1996) and approved by the Office of Research and Integrity Assurance at Georgia Tech.

## Author Contributions

The study was designed by SI and JL. Analytic calculations and numerical computations were done by SI with help from FF. SI, CH, and FF performed the experiments using the hardware designed and built by SI. The data was processed by SI, who wrote the manuscript with editorial inputs from other authors.

### Conflict of Interest Statement

The authors declare that the research was conducted in the absence of any commercial or financial relationships that could be construed as a potential conflict of interest.
