# Supplementary material for: Theoretical Modeling and Experimental Detection of the Extracellular Phasic Impedance Modulation in Rabbit Hearts
Source: Front Physiol. 2019 Jul 9;10:883. doi: 10.3389/fphys.2019.00883 (PMC6629904; doi:10.3389/fphys.2019.00883)
Supplement: Supplementary file 1 [file Data_Sheet_1.pdf]

Theoretical Modeling and Experimental Detection of the Extracellular Phasic Impedance Modulation in Rabbit Hearts

Shahriar Iravanian, MD, Conner Herndon, Jonathan J Langberg, MD, Flavio H. Fenton, PhD

1 SUPPLEMENTS

1.1 Supplement A

In this supplement, we derive the general formula for the frequency-dependent impedance of an isolated myocyte (or a patch of sarcolemma) measured using an intracellular electrode. We closely follow Bialek in the ensuing derivation (Bialek, 2012).

The model is described by Equations (1) and (2), which are reproduced here,

$$C\frac{dV}{dt} = -I(V, f_1, f_2, \ldots, f_n; \mathbf{P}) + I_{\text{ext}}\,, \tag{A1}$$

and,

$$\frac{df_i}{dt} = -\frac{1}{\tau_i(V)}\left[f_i - f_i^{eq}(V)\right]\,. \tag{A2}$$

Admittance, the inverse of impedance, is measured by calculating the ratio of current response to a small transmembrane potential perturbation δV. We linearize Equation (A1) by substituting $V + \delta V$ for V, expanding as a Taylor series, and keeping only the linear terms.

$$C\frac{d\delta V}{dt} = -\frac{\partial I}{\partial V}\delta V - \sum_i \frac{\partial I}{\partial f_i}\delta f_i + \delta I_{\text{ext}} \tag{A3}$$

In order to solve Equation (A3), we need to express δf_i is term of δV. We linearize Equation (A2),

$$\frac{d\delta f_i}{dt} = -\frac{1}{\tau_i(V)}\left[\delta f_i - \left.\frac{df_i^{eq}(V)}{dV}\right|_{V=V_0}\delta V\right]\,, \tag{A4}$$

where V_0 is the actual value of the transmembrane potential at each point in time, in contrast to the perturbed value caused by the test signal. The presence of δf_i on the right of Equation (A4) prevents a closed algebraic solution. Rather, we need to use Fourier transformation to solve this problem. For an angular velocity ω, we assume $\delta V = Ae^{-i\omega t}$. Under the linearity assumption, $\delta f = Be^{-i\omega t}$, where B is a complex number and incorporates both amplitude and phase information.

Substituting $\delta V = Ae^{-i\omega t}$ in Equation (A4),

$$-i\omega\delta\mathcal{F}_i(\omega) = -\frac{1}{\tau_i(V)}\left[\delta\mathcal{F}_i(\omega) - \left.\frac{df_i^{eq}(V)}{dV}\right|_{V=V_0}\delta\mathcal{V}(\omega)\right], \tag{A5}$$

where $\mathcal{F}_i$ and $\mathcal{V}$ are the Fourier transformation of f_i and V, respectively. Simplifying,

$$\delta\mathcal{F}_i(\omega) = \frac{1}{1 - i\omega\tau_i(V)}\left.\frac{df_i^{eq}(V)}{dV}\right|_{V=V_0}\delta\mathcal{V}(\omega)\,. \tag{A6}$$

Substituting $\delta\mathcal{F}_i(\omega)$ in Equation (A3), we obtain

$$-i\omega C\delta\mathcal{V}(\omega) = \left(-\frac{\partial I}{\partial V} - \sum_i\left(\frac{1}{1 - i\omega\tau_i(V)}\frac{df_i^{eq}(V)}{dV}\frac{\partial I}{\partial f_i}\right)_{V=V_0}\right)\delta\mathcal{V}(\omega) + \delta\mathcal{I}_{\text{ext}}(\omega) \tag{A7}$$

After rearrangement, we find the general equation for admittance,

$$\frac{1}{Z(\omega)} = \frac{\delta\mathcal{I}_{\text{ext}}(\omega)}{\delta\mathcal{V}(\omega)} = \frac{\partial I}{\partial V} - i\omega C + \sum_i\left(\frac{1}{1 - i\omega\tau_i(V)}\frac{df_i^{eq}(V)}{dV}\frac{\partial I}{\partial f_i}\right)_{V=V_0} \tag{A8}$$

Equation (3) in the main text is the same as Equation (A8), where the $V = V_0$ subscript is implicit.

1.2 Supplement B

Our goal in this supplement is to calculate the extracellular impedance and its phasic modulation. In order to do so, we need to use a steady-state bidomain model. In general, solving bidomain models require solving the resulting partial differential equations numerically. However, if certain simplifying pre-conditions are met, closed solutions are available. One such solvable problem is the voltage response of a uniform and isotropic medium to a point current source.

Assume we have a circular shaped two-dimensional cardiac tissue with a current source I_0 at the center. Our goal is to calculate the impedance by finding the extracellular voltage response to this current source.

The two-dimensional steady-steady bidomain equations are

$$\sigma_i\Delta V_i - (\beta/R_m)V_m = 0\,, \tag{B1}$$

and,

$$\sigma_e\Delta V_e + (\beta/R_m)V_m = I_0\,\delta(x)\delta(y)\,, \tag{B2}$$

where V_i and V_e are the intracellular and extracellular potentials, respectively, and $V_m = V_i - V_e$ is the transmembrane potential (all in mV). I_0 is the injection current into the extracellular space at the origin and δ is the Dirac delta function. Note that for two-dimensional problems, the unit of I_0 is mA/cm and not mA.

The intracellular and extracellular conductance are depicted by σ_i and σ_e. The membrane surface-to-volume ratio is β. In this paper, we have used the following values: $\sigma_i = 3 \times 10^{-3}\, S/cm$, $\sigma_e = 6 \times 10^{-3}\, S/cm$, and $\beta = 2000/cm$ (Plonsey and Barr, 1982; Sepulveda et al., 1989).

R_m is the membrane resistivity (in $\Omega \cdot cm^2$). It provides a link between the active ionic models and the passive bidomain model. For each time-point, we calculate the impedance measured by an intracellular electrode, Z, using Equation (3) in the body of the paper, and set R_m equal to $|Z|$ (properly scaled to $\Omega \cdot cm^2$). This process is valid because we are interested in a steady-state response, as the test signal is an unmodulated sinusoidal wave.

Equations (B1) and (B2) have an analytic solution for a uniform and isotropic media with a single point current injection (Sepulveda et al., 1989). First, we calculate the space constant as

$$\lambda = \sqrt{\frac{R_m}{\beta(1/\sigma_i + 1/\sigma_e)}}\,. \tag{B3}$$

Next, we can obtain the transmembrane potential as

$$V_m = \frac{I_0}{2\pi\sigma_e} K_o(r/\lambda)\,, \tag{B4}$$

where K_0 is a modified Bessel function of the second kind.

The next step is to transform equations (B1) and (B2) into the polar coordinate by replacing the Laplacian by its polar form. By symmetry, any derivative with respect to the angle is zero; hence, the problem reduces to an ordinary differential equation,

$$\frac{1}{r}\frac{d}{dr}(r\frac{dV_i}{dr}) - \frac{\beta}{\sigma_i R_m} V_m = 0\,. \tag{B5}$$

We solve equation (B5) numerically to find V_i. Then, V_e is derived as $V_e = V_i - V_m$. Finally, the extracellular impedance (in $\Omega \cdot cm$) is

$$\mathbb{Z} = \frac{V_e}{I_0}\,. \tag{B6}$$

1.3 Supplement C

This supplement describes the signal processing done on the voltage signal to obtain impedance based on the Lomb-Scargle spectrogram (Lomb, 1976; Scargle and D., 1982). The input voltage signal was split into non-overlapping segments of length N. In this paper, we chose $N = 300$, equivalent to 10 ms windows. Impedance was calculated for each segment separately. Let $\mathbf{y} = y_1, \ldots, y_N$ be the input signal for the current segment with the corresponding test signal phases of $\phi = \phi_1, \ldots, \phi_N$. Here, $\phi_j = \omega j \Delta t + \phi_0$, where ω is the angular frequency, Δt is the sampling interval ($\Delta t = 33.3\,\mu$sec in this paper), and ϕ_0 is the phase of the first point of the segment.

To the first approximation, we can calculate the signal power at ω as

$$P(\omega) \propto \sum_{i=1}^{N} (y_i \sin\phi_i)^2 + \sum_{i=1}^{N} (y_i \cos\phi_i)^2 \,. \tag{C1}$$

This equation is valid as long as $\sum_i \sin\phi_i \cos\phi_i \approx 0$. This holds if $N \gg 2\pi/\omega$. However, this condition does not necessarily apply for the parameters we have used in this study. Therefore, we use the Lomb-Scargle correction by shifting the phase by θ. The main idea is to chose θ in such a way to ensure $\sum_i \sin(\phi_i - \theta)\cos(\phi_i - \theta) = 0$. This is achieved by setting θ as

$$\theta = \frac{1}{2} \arctan \frac{\sum_i \sin 2\omega t}{\sum_i \cos 2\omega t} \,. \tag{C2}$$

$P(\omega)$ is then calculated as

$$P(\omega) = \frac{1}{2} \left(\frac{\sum_i (y_i \sin(\phi_i - \theta))^2}{\sum_i \sin^2(\phi_i - \theta)} + \frac{\sum_i (y_i \cos(\phi_i - \theta))^2}{\sum_i \cos^2(\phi_i - \theta)} \right) \,. \tag{C3}$$

The impedance is $|Z(\omega)| = kP(\omega)^{1/2}$, where k is a calibration factor.

REFERENCES

Bialek, W. S. (2012). *Biophysics : searching for principles* (Princeton University Press)

Lomb, N. R. (1976). Least-squares frequency analysis of unequally spaced data. *Astrophysics and Space Science* 39, 447–462. doi:10.1007/BF00648343

Plonsey, R. and Barr, R. (1982). The four-electrode resistivity technique as applied to cardiac muscle. *IEEE transactions on bio-medical engineering* 29, 541–6

Scargle, J. D. and D., J. (1982). Studies in astronomical time series analysis. II - Statistical aspects of spectral analysis of unevenly spaced data. *The Astrophysical Journal* 263, 835. doi:10.1086/160554

Sepulveda, N., Roth, B., and Wikswo, J. (1989). Current injection into a two-dimensional anisotropic bidomain. *Biophysical Journal* 55, 987–999. doi:10.1016/S0006-3495(89)82897-8
